# Use of artificial intelligence for the prediction of lymph node metastases in early-stage colorectal cancer: systematic review

**DOI:** 10.1093/bjsopen/zrae033

**Published:** 2024-04-18

**Authors:** Nasya Thompson, Arthur Morley-Bunker, Jared McLauchlan, Tamara Glyn, Tim Eglinton

**Affiliations:** Department of Surgery, University of Otago, Christchurch, New Zealand; Department of Pathology and Biomedical Science, University of Otago, Christchurch, New Zealand; Department of Surgery, Te Whatu Ora – Health New Zealand Waitaha Canterbury, Christchurch, New Zealand; Department of Surgery, University of Otago, Christchurch, New Zealand; Department of Surgery, Te Whatu Ora – Health New Zealand Waitaha Canterbury, Christchurch, New Zealand; Department of Surgery, University of Otago, Christchurch, New Zealand; Department of Surgery, Te Whatu Ora – Health New Zealand Waitaha Canterbury, Christchurch, New Zealand

## Abstract

**Background:**

Risk evaluation of lymph node metastasis for early-stage (T1 and T2) colorectal cancers is critical for determining therapeutic strategies. Traditional methods of lymph node metastasis prediction have limited accuracy. This systematic review aimed to review the potential of artificial intelligence in predicting lymph node metastasis in early-stage colorectal cancers.

**Methods:**

A comprehensive search was performed of papers that evaluated the potential of artificial intelligence in predicting lymph node metastasis in early-stage colorectal cancers. Studies were appraised using the Joanna Briggs Institute tools. The primary outcome was summarizing artificial intelligence models and their accuracy. Secondary outcomes included influential variables and strategies to address challenges.

**Results:**

Of 3190 screened manuscripts, 11 were included, involving 8648 patients from 1996 to 2023. Due to diverse artificial intelligence models and varied metrics, no data synthesis was performed. Models included random forest algorithms, support vector machine, deep learning, artificial neural network, convolutional neural network and least absolute shrinkage and selection operator regression. Artificial intelligence models’ area under the curve values ranged from 0.74 to 0.9993 (slide level) and 0.9476 to 0.9956 (single-node level), outperforming traditional clinical guidelines.

**Conclusion:**

Artificial intelligence models show promise in predicting lymph node metastasis in early-stage colorectal cancers, potentially refining clinical decisions and improving outcomes.

**PROSPERO registration number:**

CRD42023409094.

## Teaser

A comprehensive search was performed of papers that evaluated the potential of artificial intelligence in predicting lymph node metastasis in early-stage colorectal cancer. Models included random forest algorithms, support vector machine, deep learning, artificial neural network, convolutional neural network and least absolute shrinkage and selection operator regression. Artificial intelligence models’ area under the curve values ranged from 0.74 to 0.9993 (slide level) and 0.9476 to 0.9956 (single-node level), outperforming traditional clinical guidelines. Artificial intelligence models show promise in predicting lymph node metastasis in early-stage colorectal cancer, potentially refining clinical decisions and improving outcomes.

## Introduction

Colorectal cancer (CRC) is a leading cause of cancer-related death worldwide, and with the introduction of population-based screening programmes, there has been an increase in the diagnostic incidence of early (T1 and T2) CRC^1,2^. The presence of lymph node metastases (LNM) serves as a crucial prognostic indicator in determining if patients with early-stage CRC require additional surgical intervention following endoscopic resection, and if adjuvant chemotherapy is indicated after surgical resection for patients with advanced-stage disease^3–5^.

Current nodal status evaluation in patients with CRC relies on radiological imaging data such as magnetic resonance imaging or computed tomography (CT), and histopathological examination of endoscopic biopsies^6–8^. Several histologic risk factors have been proposed as predictors of regional LNM, including the presence of lymph vascular invasion, tumour budding, deep submucosal invasion and poorly differentiated cell clusters^9–11^. However, qualitative evaluation of pathological features coupled with interobserver variability among pathologists^12^, renders this approach inadequate for accurately predicting LNM in patients with CRC. In order to overcome these issues, a more reproducible and accurate prediction tool needs to be developed.

Artificial intelligence (AI) techniques including machine learning (ML) algorithms such as random forest (RF) decision trees and support vector machines (SVMs), as well as deep learning (DL) models, like convolutional neural networks (CNN), have emerged as promising avenues to improve oncological diagnosis and prognosis^13–18^. Specifically, using computer vision (CV) models such as CNN to analyse whole slide images (WSI) enables an in-depth evaluation of tissue morphology through automated feature extraction^19^. In comparison, ML algorithms assess human-derived factors such as clinical and histological variables to make their predictions. As AI-powered tools have the ability to process vast amounts of intricate data and identify hidden patterns, they have the potential to offer more accurate predictions of LNM than conventional methods. In recent years, the application of AI in cancer research has witnessed exponential growth, with research focusing on harnessing its potential to improve clinical management and patient outcomes^20–22^. The decision to proceed with colon resection after endoscopic removal of early CRC is a pivotal step. A more accurate prediction of LNM using these AI techniques can significantly guide this decision, aiming to reduce both the rate of unnecessary resections and the risk of patients developing metastatic disease due to not undergoing resection when it would have been beneficial.

The primary aim of this systematic review was to provide a comprehensive synthesis of the current state of the applications of AI in predicting LNM in early-stage (T1 and T2) CRC. Secondary outcomes included identifying variables that influenced the performance of these models and exploring the potential strategies utilized to address or mitigate these challenges.

## Methods

This systematic review was conducted and reported in line with PRISMA (Preferred Reporting Items for Systematic Reviews and Meta-Analyses, *Supplementary materials*)^23^. The protocol was registered with PROSPERO (The International Prospective Register of Systematic Reviews) (registration number: CRD42023409094).

### Search strategy and study selection

All publications were identified through a systematic search of the following five databases: PubMed, MEDLINE, Embase, Cumulative Index to Nursing and Allied Health Literature (CINAHL) and Cochrane Central Register of Controlled Trials. Search strategies for this review combined search terms that included (artificial intelligence OR neural networks OR machine learning OR deep learning) AND (colorectal neoplasms OR colorectal cancer) AND (lymph nodes OR lymph node metastases) using a combination of subject headings and keywords in the title or abstract (*Supplementary materials*). Boolean operators AND/OR connected the search terms. The initial search strategy was developed for use in MEDLINE and was then further adapted to be utilized in the other databases.

Original studies that reported the use of AI for the prediction of LNM in early-stage CRC in patients older than years of age were included. All articles published up until March 2023 were included and publications that were abstract only or not written in the English language were excluded. Further studies were excluded if participants had more than one malignant polyp or other synchronous colorectal malignancies or had a previous history of CRC. The reference lists of the manuscripts identified using the search strategies were reviewed for studies that were not captured in the initial search. All search strategies were carried out on 2 August 2023. Search records were exported into Covidence (Covidence systematic review software, Veritas Health Innovation, Melbourne, Australia) for removal of duplications and subsequent screening.

### Outcomes of interest

The primary outcome of the review was to summarize the different AI models utilized to predict LNM in early-stage CRC and describe the accuracy of these models. Secondary outcomes included identifying variables that influenced the performance of these models and exploring the potential strategies utilized to address or mitigate these challenges.

#### Data extraction

Two independent reviewers performed the first screening of publications, reviewing titles and abstracts. The records that were accepted in the initial screen were further reviewed using the manuscript full texts based on the eligibility criteria.

Two reviewers critically appraised the studies, and the quality of the studies was evaluated using the Joanna Briggs Institute (JBI) critical appraisal tools. One reviewer collected the data from the studies.

All disagreements were resolved by discussion and a consensus was reached.

#### Data analysis

As there was variability among the AI software utilized to assess LNM and some of the outcome measures were qualitative, a quantitative synthesis was not performed. The results were tabulated, and the outcomes were summarized. Each study was assessed according to its design, year, location, number of participants, AI program utilized, accuracy of the AI model and factors that influenced the reliability of the model. A critical appraisal of each manuscript was undertaken using the JBI tool which assesses the quality and applicability of studies by systematically reviewing the bias of the methodology and subsequent analyses.

## Results

### Search results

**Fig. 1:**
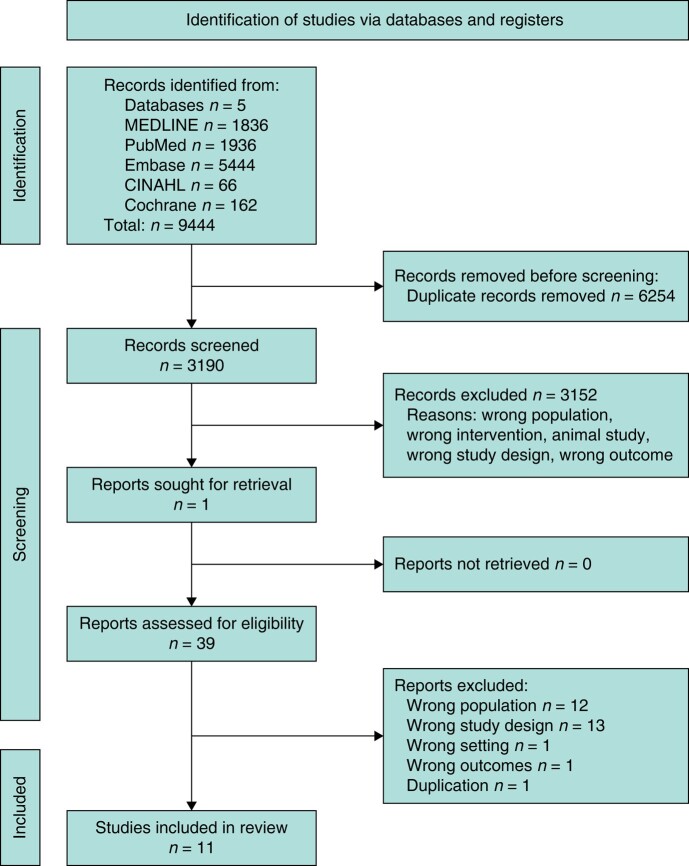
**PRISMA diagram** CINAHL, Cumulative Index to Nursing and Allied Health Literature.

After the removal of duplications, our search strategy yielded a total of 3190 citations for abstract and title review, resulting in 39 full-text reviews, of which 11 were eligible and included in the final analysis (*Fig. 1*).

All 11 studies were retrospective by design; three of these studies were single-centre^24–26^ and the other eight were multicentre cohort studies^27–34^. The collection intervals of the nine studies ranged from 1996 to 2023, with a median range of 2001 to 2016. The total number of patients across all studies was 8648, with the largest study involving 4073^30^ patients and the smallest involving 146 patients^29^. The largest study was undertaken in Japan, while the smallest was conducted in Taiwan. The studies were conducted in a variety of countries, including Japan, Taiwan, South Korea, Denmark and the USA; it is worth noting that some of the studies were conducted in non-English speaking countries.

Seven studies included patients who underwent surgical resection^24,27,28,30,32,33^, while three studies included patients who underwent endoscopic resection^29,31,33^ and one study included both^25^. In terms of cancer stage, eight studies exclusively included patients with T1 cancer, one study exclusively included patients with T2 cancer, and two studies included both T1 and T2 cancers, *Table 1*.

**Table 1:** Study characteristics

Authors	Year	Type of study	Study population (demographics, tumour stage, treatment)	Geographical distribution	Aim
Song *et al.*	2022	Retrospective multicentre cohort study	*n* = 400 patients with T1 CRC who underwent endoscopic treatment from 1996 to 2018	South Korea	The aim of this study was to determine the utility of an AI with DL of haematoxylin and eosin-stained endoscopic resection specimens without manual-pixel-level annotation for predicting LNM in T1 CRC
Takamatsu *et al.*	2022	Retrospective multicentre cohort study	*n* = 787 patients with T1 CRC endoscopically or surgically resected between 2005 and 2015	Japan	The aim of this study was to develop and evaluate a machine learning model for predicting LNM of T1 CRC without histologic assessment
Takashina *et al.*	2023	Retrospective single-centre cohort study	*n* = 585 patients with T1 and T2 CRCs endoscopically or surgically resected between 2001 and 2018	Japan	The aim of the study was to develop a novel AI model for predicting LNM using WSI in T1 CRCs and evaluate its performance
Brockmoeller *et al.*	2022	Retrospective single-centre cohort study	*n* = 514 (pT2: 311, pT1: 203) CRC patients with bowel resections and known LN status. pT2 cohort from Køge/Roskilde and Slagelse hospitals (2010–2014), pT1 cohort from a nationwide study of malignant colorectal polyps (2001–2011)	Denmark	The aim of this study was to use DL to predict the presence of LNM and whether more than one of the examined LN was reported as being positive
Kwak *et al.*	2020	Retrospective multicentre cohort study	*n* = 164 patients diagnosed with T1 and T2 CRCs excluding those with rectal cancer. Based on the AJCC staging system, patients were divided into LNM-positive (stage III) and LNM-negative (stage I and stage II) groups	Japan	To predict the presence of LNM from histopathological features in patients with T1 CRCs using a deep CNN to identify a pathological parameter for reliable accurate assessment of LNM using a CNN model that can better stratify patients with CRC
Ichimasa *et al.*	2022	Retrospective multicentre cohort study	*n* = 511, pT2 CRC who underwent surgical resection with LN dissection. 411 were assigned to the training cohort and 100 to the validation cohort from 2005 to 2015	Yokohama, Japan	The aim of the study was to develop and validate a simple, non-invasive tool for accurately predicting LNM in patients with T2 CRC using data obtained after endoscopic resection to help triage patients who may require additional surgery
Kang *et al.*	2020	Retrospective multicentre cohort study	*n* = 381, T1 CRC who underwent surgical resection from 2004 to 2011	Korea	The aim of this study was to investigate the predictive value of IHC results of TILs in addition to routinely reported pathologic parameters
Kasahara *et al.*	2022	Retrospective multicentre cohort study	*n* = 146, T1b CRC biopsy specimens, 1996–2018	Tokyo, Japan	The aim of the study was to develop a machine learning model to predict LNM risk in T1 CRC using morphological and quantitative features of cancer cell nuclei
Kudo *et al.*	2020	Retrospective multicentre cohort study	*n* = 4073 patients with T1 CRC, who underwent endoscopic resection alone, initial or additional surgical resection with LN dissection from April 1997 to September 2017	Japan	The aim of this study was to develop an algorithm to predict LNM in patients with T1 CRCs. Moreover, to utilize external validation to assess the usefulness as a decision-making tool with respect to the need for additional surgical resection after endoscopic resection
Takamatsu *et al.*	2019	Retrospective multicentre cohort study	*n* = 397 T1 CRC surgically or endoscopically resected between 2005 and 2012	Japan	This study aimed to develop a machine learning-based LNM prediction model for T1 CRC without conventional histologic assessment
Ichimasa *et al.*	2018	Retrospective single-centre cohort study	690 patients who had been diagnosed as having T1 CRCs and had undergone resection either endoscopically or surgically from April 2001 to March 2016	Japan	The aims of this study were to use AI to develop a high-performance prediction model for LNM in pT1 CRCs, to quantify the associated reductions in unnecessary additional surgery and to compare the accuracy of prediction of LNM with those of the NCCN, ESMO and JSCCR guidelines

CRC, colorectal cancer; AI, artificial intelligence; DL, deep learning; LNM, lymph node metastases; WSI, whole slide imaging; LN, lymph node; AJCC, American Joint Committee on Cancer; CNN, convolutional neural network; IHC, immunohistochemistry; TILs, tumour-infiltrating lymphocytes; NCCN, National Comprehensive Cancer Network; ESMO, European Society for Medical Oncology; JSCCR, Japanese Society for Cancer of the Colon and Rectum.

### Quality of the studies

The quality of all the studies was evaluated using the JBI scoring checklists specific to each study design. The studies were rated based on the number of fulfilled criteria, excluding criteria that were not relevant to the study. All of the studies included in the review satisfied a high proportion of criteria ranging from 87.5 to 100%, which suggests a low level of risk of bias. The results of these assessments can be seen in the *Supplementary materials*.

### AI algorithms and models used

The 11 studies reviewed employed various AI models and approaches for predicting cancer prognosis. These fell into two distinct categories, those that used CV-based assessment of histopathological images (*Table 2*) and the remainder employed AI analysis of clinicopathological features (*Table 3*). Techniques such as attention-based DL and CNNs, alongside feature extraction from WSIs, enable precise integration of clinical variables and image-based features, streamlining the diagnostic process. In the studies that utilized AI for direct analysis of histopathological images, CNN were predominantly employed for metastasis detection or for classifying cancerous regions by histologic type^31–34^. Preprocessing steps in these studies included data augmentation^28,30^ and techniques like segmentation of cell nuclei and pixel thresholding to analyse large histopathological images. Attention-based DL models are used to rank the relative importance of tissue regions in a haematoxylin and eosin WSI and aggregate information for making a prediction^35^. Preprocessing steps include normalization of features to increase classification accuracy.

**Table 2:** Artificial intelligence algorithms and models

Image analysis using a CV algorithm			
Authors	AI models used	Features and variables used by these models (for example clinical, histopathological and radiological data)	Preprocessing steps (for example data normalization, feature selection or augmentation)
Song *et al.,* 2022	The AI model employed in this study utilized a two-step approach of attention-based DL. This was achieved by training a DCNN at the patch level to extract features from histopathological images and construct an end-to-end neural network for training a WSI-level classifier. Additionally, this model used an AM to leverage the relative importance of each patch image that contributed to the classification task	The clinical features and variables used by this model included age at diagnosis, sex, body mass index, presence of co-morbidity, family history of CRC, smoking status, alcohol consumption, endoscopic morphology of the tumour and tumour location, as well as pathologic features such as size of cancer, histologic differentiation, depth of SM invasion, LVI, tumour budding, positive resection margin and microsatellite instability. Assessment of histological differentiation was based on the least high-grade histologic pattern	Preprocessing steps included patch-level tiling of WSIs for input to the DCNN FE, compression and encoding of each patch image into a FV, and aggregation of the FVs into a single WSI-level FV by their weighted average, with attention score computed through the AM. After DL extraction from these WSIs, they were then concatenated with the patients’ clinical features to formulate the final FV for each patient. This was then fed into the classification layer to obtain the final prediction
Takamatsu *et al.,* 2022	The AI models employed in this study included a CNN for image classification and a RFC for LNM prediction	The data contained 18 parameters as follows: tumour location; total number of cancer-class tiles; number of tiles classified as metastatic or non-metastatic; number of Group A, B, D, E tiles; percentages of tiles classified as metastatic or non-metastatic; average probabilities; standard deviations of cancer-class probabilities and metastatic or non-metastatic probabilities; and probability score summary for each tile. The RF algorithm randomly collected these parameters to create decision tress, and the importance of each parameter was averaged in all the trees	The WSIs were cut into non-overlapping small tile images of 299-pixel squares (equal to a 273-μm square). To ensure that the image contained at least one nucleated cell, each tile image was temporarily converted to grey scale (0–255) and the image containing the minimum pixel value of 110 was adopted for further image analysis
Takashina *et al.,* 2023	The study used a deep CNN architecture called Xception to pretrain the CNN and extract numerical features from the pathological patch images. Unsupervised training was conducted using K-means, which is a non-hierarchical clustering method. A RF algorithm was used to train the extracted features for LNM prediction	To extract features from each WSI patch images were classified into 10 clusters. Next, the ratio of the number of each cluster was calculated (10 features from one WSI). Patients’ sex (female, 1; male, 1) and cancer location (rectum, 0; colon, 1) were also extracted as features	The captured whole slide images were divided into 224-pixel square patches, and blank patches and patches without cancerous areas were excluded. Pretraining was performed using 5000 pathological patch images from the Kather database
Brockmoeller *et al*., 2022	Deep neural network models trained on haematoxylin and eosin whole slide images. ShuffleNet network model with transfer learning for end-to-end prediction of LNM status	Presence of LNM status, more than one of the examined LN being positive, dMMR status directly from the haematoxylin and eosin whole slide image. Pedunculated and sessile pT1 CRC subgroups	Data preprocessed according to the ‘Aachen protocol for DL histopathology’. Tiles of 512 × 512 pixels extracted. Tiles containing background or artefacts removed based on canny edge detection. Colour normalization using Macenko normalization
Kwak *et al.,* 2020	Deep CNN based on the U-Net architecture	Histopathological images of CRC with seven tissue classes: normal colon mucosa, stroma, lymphocytes, mucus, adipose tissue, smooth muscle and colon cancer epithelium	All images were normalized using the Macenko method and were preprocessed prior to thresholding via histogram normalization
Kasahara *et al.,* 2022	The study employed machine learning algorithms, including SVM and RF. A model was also constructed for DL to recognize the nucleus based on its long axis	The study used morphological and quantitative features of cancer cell nuclei, including size, roundness, contour line length, major and minor axis length, eccentricity, perimeter, solidity, chromatin texture-related features such as contrast, entropy, second angular moment, and heterogeneity/pleomorphism quantified using co-occurrence matrix and Haralick functions	Nuclei were extracted using the Ilastik software package and the final morphological and quantitative measurements of cell nuclei were performed using the CellProfiler software package

CV, computer vision; AI, artificial intelligence; DL, deep learning; DCNN, deep convolution neural network; WSI, whole slide imaging; AM, attention module; CRC, colorectal cancer; SM, submucosal; LVI, lymphovascular invasion; FE, finite element; FV, feature vector; CNN, convolutional neural network; RFC, random forest classifiers; LNM, lymph node metastases; RF, random forest; LN, lymph node; dMMR, mismatch repair deficient; SVM, support vector machines.

**Table 3:** Artificial intelligence algorithms and models

AI analysis of clinicopathological features			
Authors	AI models used	Features and variables used by these models (for example clinical, histopathological and radiological data)	Preprocessing steps (for example data normalization, feature selection or augmentation)
Ichimasa *et al.,* 2022	RF algorithm for machine learning	The prediction model used eight variables: patient age, patient sex, tumour size, tumour location, lymphatic invasion, vascular invasion, histologic differentiation and serum CEA level. Clinical factors (patient age and sex, tumour location and size, and serum CEA level) were extracted from the electronic health record system. Tumour location was dichotomized as one of two colonic segments (caecum or ascending to the sigmoid colon) or rectum. Tumour size was measured after formalin fixation. CEA was measured before surgical resection	The study did not explicitly describe any preprocessing steps, such as data normalization, feature selection or augmentation
Kang *et al.,* 2020	LASSO regression	Clinicopathologic variables including depth of invasion, histologic grade, tumour budding, LVI, and IHC MMR status and TILs, were utilized in the prediction models	Preprocessing steps were not explicitly mentioned in the text, but feature selection using LASSO regression was performed to identify the most important variables for the prediction model. Cut-off values for IHC markers were also determined using Youden's index and accuracy
Kudo *et al.,* 2020	The study employed an ANN model using a feed-forward network	The following features and variables were used by the ANN model: patients' age, sex, tumour size, location, morphology, lymphatic invasion, vascular invasion and histologic grade	Data normalization was performed on age and tumour size, which were normalized to be in a range (0,1) for classification accuracy
Takamatsu *et al.,* 2019	The study employed a RFC machine learning algorithm	The largest slice of each tumour was selected for evaluation of the following histologic factors: tumour depth, lymphatic invasion, venous invasion, poorly differentiated clusters and tumour budding	Preprocessing steps included image resolution equalization, deletion of non-cancerous areas, binary image conversion and morphological analyses
Ichimasa *et al.*, 2018	SVM	45 clinicopathological factors including lymphatic invasion, vascular invasion, histological features, lactase dehydrogenase and others as listed in their *Table 1*	Data was divided into training and validation sets based on date. The importance of each factor used in the AI model was calculated

AI, artificial intelligence; RF, random forest; CEA, carcinoembryonic antigen; LASSO, least absolute shrinkage and selection operator; IHC, immunohistochemical; MMR, markers for mismatch repair; TILs, tumour-infiltrating lymphocytes; ANN, artificial neural network; RFC, random forest classifiers; SVM, support vector machines.

In the study conducted by Brockmoeller *et al.*^24^ the researchers employed deep neural network models specifically trained on haematoxylin and eosin-stained WSI to predict LNM in patients with CRC. They utilized the ShuffleNet network model, which is known for its efficiency and accuracy in image classification tasks. They also used transfer learning, a technique that leverages pretrained models on a new but related task. This approach was used to reduce the time required to train the model and the data requirements, which is especially beneficial when dealing with medical images where data can be scarce. The study aimed to predict various parameters directly from the WSI, such as the presence of LNM status, the number of examined LN being positive, and the DNA mismatch repair status (dMMR). They also distinguished between different subgroups of CRC, namely pedunculated and sessile pT1 CRC. To ensure the quality and consistency of the input data, the images underwent a rigorous preprocessing regimen. This involved extracting tiles of 512 × 512 pixels in size from the WSIs and removing tiles containing background or artefacts based on Canny edge detection. Furthermore, to address the common issue of colour variations in histopathological images, they employed the Macenko colour normalization method, ensuring consistent colour representation across all images.

Other studies utilized ML techniques that incorporated both clinical and histopathological features. Random forest classifiers (RFC) and DL models were commonly used in this category^36^. Takamatsu *et al.*^32^ used a supervised ML approach to predict LNM based on 16 parameters in a data set of 397 patients with CRC. The data set was randomly split into a training set (70%, *n* = 277) and a test set (30%, *n* = 120) with similar LNM rates. The RFC ML algorithm was selected to minimize overfitting^32^. The RFC then creates a set of decision trees from randomly selected parameters of the training data set; the computer-learned patterns were then used to predict cases as positive or negative for LNM.

Kudo *et al.* developed a predictive model using eight factors: patient age, sex, tumour size, tumour location, morphology, lymphatic invasion, vascular invasion and histological grade^34^. An artificial neural network (ANN) then utilized this model to predict the likelihood and risk of LNM metastases. ANN models are comprised of various interconnected neurons that share information and each neuron is weighted at a different level of significance which can be updated through a training process^37,38^. This allows the ANN to continually develop relationships between input and output variables. The network was trained by iteratively changing the class weight and a hyperparameter evaluation was performed to obtain the optimum parameter set.

### Model performance metrics

*Tables 4* and *5* summarize the performance metrics that were utilized by the studies to evaluate the accuracy of the AI models and the comparative analyses employed to assess their models’ performance against traditional methods. The performance metrics such as area under the curve (AUC), sensitivity, specificity and accuracy were employed across studies to evaluate AI models. Comparatively, AI models were assessed against traditional methods, with certain studies noting AI's capability of enhancing predictive precision while potentially reducing the need for unnecessary interventions, as indicated by specific comparisons and statistical analyses. Kasahara *et al.* also demonstrated high levels of accuracy utilizing a regions of interest (ROI)-based discrimination of LNM risk, with their SVM and RF models achieving a case-by-case accuracy of 81.8% and 86.8% respectively. When validating their prediction models Kudo *et al.* and Song *et al.* observed improved specificity of their models compared with current US and Japanese Society for Cancer of the Colon and Rectum (JSCCR) guidelines, with the AI model having safely reduced 15.1% of unnecessary additional surgeries that would be indicated when using current management guidelines.

**Table 4:** Model performance and metrics

Image analysis using a CV algorithm		
Authors	Performance metrics used in the included studies to evaluate the AI models, such as sensitivity, specificity, accuracy, AUC and F1 score	Comparison of the AI models' performance to traditional methods, if applicable (for example clinical nomograms, TNM staging system)
Song *et al.,* 2022	The performance metrics used to evaluate the AI model included AUC of the ROC curve. The AUC value for predicting LNM in T1 CRC was 0.747 for the training set and 0.764 for the test set. Without clinical features their model showed better performance with an AUC of 0.747 for the training set and 0.764 for the test set compared with prediction using clinical and/or pathologic features	The validation of the AI model's performance was compared with using only clinical features, only pathological features or combined clinical and pathologic features. The model was also compared with a RFC with 500 trees to make predictions for LNM and to JSCCR guidelines using McNemar's tests. The AI model safely avoided 15.1% of unnecessary additional surgeries compared with JSCCR guidelines
Takamatsu *et al.,* 2022	Performance of case-based prediction models for both training and validation data sets was evaluated with the AUC. The AUC for predicting LNM in the training and validation sets was 0.971 and 0.760 respectively. The specificity and sensitivity were balanced when the RF score was 0.70, and the accuracy of the model for the training and validation sets was 0.91 and 0.75 respectively	548 training cases were cross-validated to 235 cases, with AUC and operating characteristics reported in the training and validation sets as 0.971 and 0.760 respectively
Takashina *et al.,* 2023	The AUC in the receiver ROC was used to evaluate the performance of the AI model. The AUC for prediction of LNM by the AI model was 0.745 (95% confidence interval (c.i.) 0.581 to 0.860)	Validation and comparison with traditional methods: the performance of the AI model was compared with the current treatment guidelines for additional resection of T1 CRC. When the guideline criteria for additional resection of T1 CRC were applied to the test cases, the AUC was 0.524 (95% c.i. 0.501 to 0.546). The AUC of the AI model was found to be statistically superior to the AUC of the guidelines (*P* = 0.0028)
Brockmoeller *et al*., 2022	The prediction of the presence of more than one LNM in all pT1 CRCs had a cross-validated AUROC of 0.733 (0.67–0.758) and patients with any LNM had an AUROC of 0.711 (0.597–0.797). The prediction of one or any LNM status in patients with pT1 had an AUROC of 0.733 (0.644–0.778) and 0.567 (0.542–0.597) respectively	A multivariate linear regression was performed to compare how DL prediction of LNM compares with established histopathological risk factors. In the pT1 cohort, the AI score (normalized between 0 and 1) and ‘venous invasion’ (with values 0 and 1) achieved high coefficients for the presence of LNM (*b* = 0.35, *P* = 0.07 and *b* = 0.28, *P* < 0.001 respectively). In the pT2 cohort, only differentiation grade and tumour location were available. Combined with these factors in a multivariate model, the AI score achieved the highest coefficient and a clear statistical significance (*b* = 0.82, *P* < 0.001)
Kang *et al.,* 2020	The AUROC values for the individual TIL subtypes ranged from 0.50 to 0.59, while the LASSO model had an AUROC of 0.795 in the training set and 0.765 in the validation set	Performance of this model, in comparison to Japanese criteria, was measured with the AUROC analysis (0.765 in the LASSO model *versus* 0.518 in the Japanese criteria, *P* = 0.003), net reclassification improvement (0.447, *P* = 0.039), integrated discrimination improvement calculation (0.121, *P* = 0.034) and decision curve analysis (positive net benefit) within the validation set
Kasahara *et al.,* 2022	Multivariate analysis using step-wise discriminant analysis and canonical discriminant analysis, the overall accuracy rate for each ROI was 91.9% for discriminating LNM risk, and the accuracy rate for each ROI in Groups A–C (risk 0, risk 1–3 and risk 4) was 91.0%. For the machine learning models using SVM and RF, the ROI accuracy rates for the test cases of each model were 80%, 72% and 79% for Groups A–C respectively	The study validated the AI models' performance using test cases, and the accuracy rate of each ROI was 100% in the correct diagnosis rate for each training case of SVM and RF. The case-by-case accuracy rates of the test cases for each of the three models were 81.8% for SVM (e1071), 86.8% for SVM (ksvm) and RF for SVM (e1071), and 86.8% for SVM (ksvm) and RF

CV, computer vision; AI, artificial intelligence; AUC, area under the curve; TNM, tumour node metastases; ROC, receiver operating characteristic; LNM, lymph node metastases; CRC, colorectal cancer; RFC, random forest classifiers; JSCCR, Japanese Society for Cancer of the Colon and Rectum; RF, random forest; AUROC, area under the receiver operating characteristics; DL, deep learning; TIL, tumour-infiltrating lymphocyte; LASSO, least absolute shrinkage and selection operator; ROI, regions of interest; SVM, support vector machines.

**Table 5:** Model performance and metrics

AI analysis of clinicopathological features		
Authors	Performance metrics used in the included studies to evaluate the AI models, such as sensitivity, specificity, accuracy, AUC and F1 score	Comparison of the AI models' performance to traditional methods, if applicable (for example clinical nomograms, TNM staging system)
Ichimasa *et al.,* 2022	The AUC of the AI system was 0.93 within the validation data set, compared with 0.88 of the nomogram. The AI model had a sensitivity and specificity of 96% (95% c.i. 90 to 99%) and 88% (95% c.i. 80 to 94%) respectively	The AI model's performance was validated using a separate data set of 100 patients. The AUC of the AI system in the validation data set was 0.93, compared with 0.88 for the nomogram. The AI system outperformed the nomogram in terms of discriminative power
Kudo *et al.,* 2020	In the training cohort, the AUC of the ANN model was 0.78, and in the validation cohort, it was 0.83. When the target was limited to the initial endoscopic resection group (*n* = 517), the AUCs of the ANN model and the Japanese guidelines were 0.84 and 0.61 (*P* < 0.001) respectively	The performance of the ANN model was compared with that of the US guideline and the Japanese guideline. When the threshold was set to sensitivity of 79% between the ANN model and the US guideline, in the training cohort, the ANN model had a specificity of 64.7% compared with 54.7% for the US guideline. The ANN model showed higher predictive power (AUC = 0.83) than the Japanese guidelines (AUC = 0.57) for LNM in T1 CRCs (*P* < 0.001). The ANN model outperformed the Japanese guidelines in both total (AUC = 0.78 *versus* 0.57, *P* < 0.001) and the initial endoscopic resection group (AUC = 0.84 *versus* 0.61, *P* < 0.001)
Takamatsu *et al.,* 2019	The areas under the ROC curve (AUCs) were 0.938 for machine learning and 0.826 for the conventional method. The sensitivity and specificity of the optimal cut-off value for predicting LNM by machine learning were 80.0% and 94.5% respectively	Cross-validation was performed with repeated random subsampling. The average AUC of machine learning was 0.822 (0.767–0.938) and that of the conventional method was 0.855 (0.780–0.936), and there was no significant difference between them
Ichimasa *et al.*, 2018	The results indicate that the AI model using SVM (AUC = 0.821) had better discriminating power than the logistic regression model (AUC = 0.789) to identify the presence of LNM in T1 CRCs. Sensitivity was 100% (95% c.i. 72 to 100%) in all models	Specificity of the AI model and the American, European and Japanese guidelines was 66% (95% c.i. 56 to 76%), 44% (95% c.i. 34 to 55%), 0% (95% c.i. 0 to 3%) and 0% (95% c.i. 0 to 3%) respectively; and accuracy was 69% (95% c.i. 59 to 78%), 49% (95% c.i. 39 to 59%), 9% (95% c.i. 4 to 16%) and 9% (95% c.i. 4 to 16%) respectively. The rates of unnecessary additional surgery attributable to misdiagnosing LNM-negative patients as having LNM were: 77% (95% c.i. 62 to 89%) for the AI model, and 85% (95% c.i. 73 to 93%; *P* < 0.001), 91% (95% c.i. 84 to 96%; *P* < 0.001) and 91% (95% c.i. 84 to 96%; *P* < 0.001) for the American, European and Japanese guidelines respectively
Kwak *et al.,* 2020	The proposed model achieved high segmentation performance, scoring a test mean DSC of 0.892. This study observed balance class performance for all neural networks tested across other architectures. PTS score had a moderate ability to identify the presence of LNM in colon cancer (AUC 0.677; 95% c.i. 0.593 to 0.760)	No direct comparison with traditional methods provided in the results

AI, artificial intelligence; AUC, area under the curve; TNM, tumour node metastases; ANN, artificial neural network; LNM, lymph node metastases; CRC, colorectal cancer; ROC, receiver operating characteristic; SVM, support vector machine; DSC, dice similarity coefficient; PTS, predictive value of the peri-tumoral stroma.

Takamatsu *et al.* reported no significant difference in their cross-validation study, demonstrating AUCs of 0.938 and 0.826 for ML and conventional methods respectively. In a more recent study, Takamatsu *et al.* achieved comparable AUCs (training = 0.971, validation = 0.760) when incorporating CNN into an AI model to predict LNM. Takashina *et al.* found the AUCs from their model compared with traditional guidelines (0.74 (95% c.i. 0.58 to 0.86) *versus* 0.52 (95% c.i. 0.5 to 0.55)) were statistically superior (*P* = 0.0028). Ichimasa *et al.* employed an SVM model, achieving a sensitivity of 100% and specificity of 66% for predicting LNM in T1 CRC, outperforming the National Comprehensive Cancer Network (NCCN), European Society for Medical Oncology (ESMO) and JSCCR guidelines.

### Factors affecting AI model performance

Several factors were found to influence the performance of the AI models across the nine studies (*Table 6*). When reviewing input features Kang *et al.* demonstrated that tumour-infiltrating lymphocyte (TIL) subtypes, clinicopathologic parameters and the use of the LASSO algorithm for feature selection all influenced the accuracy of the prediction model^28^. In this particular study, this factor was addressed through cross-validation to optimize the hyperparameters of the LASSO algorithm. Overall, the size and quality of the training data set and the inclusion of clinicopathological features were consistently found to potentially impact AI model performance. Brockmoeller *et al.* highlighted the significance of having high-quality training data and, in particular, the relevance that the training images have to the prediction task. Their study also underscored the importance of using WSI without manual annotations, and how the subsequent choice of DL algorithm may impact model performance. By directly predicting LNM status from primary tumour slides without manual annotations, they showcased the potential of AI in histopathological analysis. Their findings suggest that future studies could delve deeper into the impact of manual annotations on model performance, explore the benefits of larger data sets and compare different DL architectures to optimize results.

**Table 6:** Factors affecting artificial intelligence model performance

Authors	Factors that were found to impact the performance of the AI models, such as the size and quality of the training data set, the choice of algorithm or the use of different input features	How these factors were addressed or could be addressed in future studies
Song *et al.,* 2022	Factors that impacted the performance of the AI model included the size and quality of the training data set and the choice of algorithm. Due to graphics processing constraints, WSIs that contained more than 1500 patch images had to be randomly downsampled to obtain only 1500 images. The use of attention-based DL and the inclusion of patient clinical and pathologic features were found to improve the model's performance	As most (98%) of WSIs contained under 1500 patch images, the sampling strategy utilized is not likely to have had significant influence on the model’s learning. To address these factors in future studies, larger and more diverse data sets could be used for training and validation, and other AI techniques such as transfer learning and ensemble methods could be explored. Additionally, the use of standardized histopathological image acquisition and processing methods could improve the quality and consistency of the data
Takamatsu *et al.,* 2022	Factors identified by the authors to impact the AI model’s performance include interobserver disagreements in histopathologic evaluations, data heterogeneity and quality from different institutional procedures, and the complexities introduced by combining CNN and RF algorithms	Several recommendations were highlighted in this study to overcome these variables, including the utilization of Grad-CAM which allows direct histologic image analyses and visualization to generate a clearer model. A multicentre validation study is needed with the addition of longer patient follow-ups to ensure a broader applicability of the prediction capability
Takashina *et al.,* 2023	It was also acknowledged that the heterogeneity of the pathological malignancy is likely to make it difficult to determine the true malignancy potential from one patch. This study also has inherent limitations being a single-centre study and did not consider tumour size	The utilization of multicentre data and validation to external data sets were recognized as key next steps. Possible utilization of more than a single pathological slide from each case
Brockmoeller *et al*., 2022	Factors recognized by the authors to impact performance included biases in tissue region selection and bias introduced during preprocessing from artefacts within the tiles	Deep neural network models were trained on all of the available hematoxylin and eosinWSIs without restriction of tissue types or the areas used. This allows an unbiased learning system with regards to tissue region in which to detect the predictive factors. To reduce the bias caused by artefacts, tiles containing these were removed based on canny edge detection in Python's OpenCV package
Kwak *et al.,* 2020	The quality of the histopathological data such as poor image quality, bad hematoxylin and eosin staining, duplicated images and artefacts were recognized to impact the data set. Additionally, the choice of algorithm and input features were also noted to influence AI performance	Exclusion of inadequate image data: slides with poor image quality, bad hematoxylin and eosin staining, duplicated images and artefacts were excluded. Use of U-Net architecture: the U-Net architecture was chosen for its ability to improve the performance of fine segmentation and localization, particularly for biomedical images
Ichimasa *et al.,* 2022	The study found that lymphatic invasion was the most influential factor for LNM among the eight examined factors	The study did not explicitly address factors that could impact the performance of the AI models, such as the size and quality of the training data set or the choice of algorithm. Future studies could address these factors by using larger and more diverse data sets, exploring different algorithms and conducting sensitivity analyses to assess the robustness of the models
Kang *et al.,* 2020	Factors that were found to impact the performance of the AI models included the choice of input features (TIL subtypes and clinicopathologic parameters) and the use of the LASSO algorithm for feature selection. The size and quality of the training data set may also have an impact on model performance	In an attempt to address these factors Kang *et al.* (2020) selected relevant clinicopathologic parameters and TIL subtypes for inclusion in the LASSO model and utilized cross-validation to optimize the hyperparameters of their algorithm. As this study used a relatively small sample size, future studies would benefit from larger data sets to further validate the performance of their AI model. Additionally, in this study, co-morbidities were not considered as potential confounding factors which could impact model performance and should look to be addressed in future studies
Kasahara *et al.,* 2022	This study found that one of their biggest limitations was the small number of centres included; different staining methods and specimen characteristics could significantly affect the results	Future studies based on prospective multicentre sites are required to validate and expand on these findings
Kudo *et al.,* 2020	The authors noted the data set was heavily imbalanced with the dependent variables. Other biases acknowledged were the use of surgically resected tissue *versus* endoscopic and the type of clinicopathological data sets being used	As the data was heavily imbalanced this study utilized a weighting regularizer to address the dependent variable imbalance and by performing a hyper-parameter evaluation to obtain the optimum parameter set. They also utilized various types of clinicopathological data using larger numbers of samples. Future studies could address these factors by using larger and higher quality data sets, comparing different algorithms and input features, and performing more rigorous validation methods
Takamatsu *et al.,* 2019	The most important parameter for predicting LNM was the Feret Y diameter. All but one training data set (92%) showed that the Feret Y diameter was the most important parameter	In an attempt to minimize the potential of overfitting, this study selected the RFC machine learning algorithm. Future studies could address the potential bias in the data set due to the retrospective nature of the study and expand the analysis to a larger, more diverse population
Ichimasa *et al.*, 2018	The inclusion of 314 patients (53%) in the training data set (*n* = 590) who had undergone primary surgery means that the AI model was not developed using only the target group, for which the model will be used	The study divided the patients into two data sets for training and validation, ensuring a separate set for model validation; this could be improved by using only endoscopic resections. The SVM algorithm was chosen based on its suitability for the data and the problem at hand. The importance of each factor used in the AI model was also calculated. Future studies could potentially benefit from larger data sets, further refinement of input features and exploration of other algorithms for improved performance

AI, artificial intelligence; DL, deep learning; WSI, whole slide imaging; CNN, convolutional neural network; RF, random forest; CAM, class activation mapping; LNM, lymph node metastases; TIL, tumour-infiltrating lymphocyte; LASSO, least absolute shrinkage and selection operator; RFC, random forest classifiers; SVM, support vector machine.

## Discussion

With the rising diagnostic incidence of early-stage (T1 and T2) CRC, accurate prediction of LNM has become increasingly critical in optimizing therapeutic strategies^3,5^. This review highlights the potential of different AI models in predicting the presence of LNM in early-stage CRC. AI prediction models may offer improved accuracy leading to earlier detection, enhanced treatment planning and overall improved outcomes for patients. However, variability among the models used, algorithm selection and input features strongly influence the performance and generalizability of the AI models.

In this review, AI models employed to predict LNM in early-stage CRC were broadly categorized into two main groups: those focusing on image analysis using CV algorithms and those centred on AI analysis of clinicopathological features. For the former group, researchers utilized DL models to analyse WSIs and histopathological data^24,26,31,34^. Brockmoeller *et al.* highlighted the ability of AI to predict LNM status from tumour slides without the addition of manual annotations. Their study underscored the significance of high-quality training data and selection of algorithms utilized^24^. Furthermore, Kwak *et al.* emphasized how poor image quality, bad haematoxylin and eosin staining, duplicated images and artefacts within the data set can influence image analysis and the subsequent choice of DL architecture selected. In the latter group, these studies predominantly employed ML techniques, such as RF algorithms, ANN and LASSO regression, focusing on the incorporation of clinicopathological variables^25,30–33^. Ichimasa *et al.* incorporated 45 clinicopathological factors in their SVM model, demonstrating the complexity and diversity of input features utilized to achieve robust predictions^25^. An advantage of this approach is that it allows clinicians to better comprehend the analysis results and enables them to provide patients with an explanation as to why the algorithm arrived at these conclusions. Having a sufficient understanding of the algorithms utilized to predict LNM is critical for the successful integration of ML in clinical medicine, benefiting both patients and clinicians in guiding well-informed treatment decision-making.

Regardless of the approach utilized, interobserver disagreements on the prediction of LNM based on histopathological risk factors have remained a challenge^39,40^; as such the diagnostic reproducibility of AI models is critical to generate a stable and reliable prediction model. Similar results were also demonstrated in the other articles included in this review^27–31^. The predictive ability of these models was ascertained through performance metrics such as the area under the receiver operating characteristic curve (AUC). Overall, the AI models were shown to demonstrate a high level of accuracy and potential for the reduction of subsequent unnecessary resections when compared with conventional guidelines^27–30^. However, the heterogeneity observed across the different study designs, patient population numbers, AI models and performance metrics utilized in the individual studies is likely to impact the generalizability of these findings.

AI models have demonstrated promising potential in improving the accuracy of LNM prediction in early-stage CRC, often outperforming traditional guidelines^27,31,41^. These differences would likely result in earlier detection, enhanced treatment planning and overall improved outcomes for patients. Specifically, the AI model utilized by Ichimasa *et al.* demonstrated a significantly higher specificity when compared with the NCCN, ESMO and JSCCR guidelines, while maintaining a sensitivity and negative predictive value (NPV) of 100%^25^. These results translated to a potential reduction in unnecessary operations by 8–14%^25^. Another study that utilized an ANN model on 3134 patients with T1 CRC achieved an AUC of 0.83 in their validation cohort, which outperformed the clinical guidelines AUC of 0.73^30^. These models highlight the potential of AI to reduce over-surgery rates and improve patient outcomes through enhanced metastasis detection in clinical settings. Despite these advantages, some of the limitations of these models include the need for large high-quality data sets that are required to train the models, ambiguity surrounding interpretability, and the possibility of the introduction of biases through the initial data collection and model development stages^27–29^. Additionally, extensive computational resources and expertise may be required to be able to successfully implement and maintain these programs.

Numerous factors were identified to influence the performance of AI models in predicting LNM, including histological subtypes, size of the lesion, algorithm selection, input features chosen, as well as the size and quality of the data set utilized to train the model. The choice of TIL subtypes^28^, incorporation of clinical parameters^30,31,33^ and utilization of the LASSO algorithm to guide feature selection also influenced the models’ overall performance. Of note, smaller sample sizes in some of the studies and recognition of particular confounding factors such as patient co-morbidities, variation in staining methods and specimen characteristics may also have an influence on the AI models’ accuracy and robustness^29^. To address these challenges, it is essential to incorporate a more diverse set of training samples, particularly to include both isolated tumour cells and underrepresented histologic subtypes. Enhancing model performance and ensuring the generalizability of these approaches necessitate further refinement of the feature selection process to allow optimization of the hyperparameters of a given algorithm. Increasing the size of quality training data sets and recognition of potential confounding factors will assist in improving the performance of these models, ultimately enhancing the reliability and accuracy of the prediction of LNM.

Future directions of the use of AI in the prediction of LNM in early-stage CRC should focus on addressing the limited sample sizes available, lack of diversity amongst the training data sets and inadequate evaluation and application of these AI models within the clinical setting. In an attempt to reduce the training time and enhance the generalizability of the models, a key area of exploration should investigate the use of transfer learning, where pretrained models are then fine-tuned on CRC-specific data sets^42^. Additionally, new methodology innovations are being developed that will be of assistance in addressing limited sample sizes and performance of AI models, for example generative adversarial networks (GANs) and vision transformers (ViTs). GANs are trained on the real original images of the sample data set, which the GANs use to synthetically produce new images that are similar in appearance to the original input images. These GANs can also be conditioned with certain attributes or labels from the original sample data set to produce synthetic images that are otherwise identical to the original sample including molecular phenotypic information^43^. ViTs are a type of neural network based on a transformer architecture which were originally utilized in natural language processing tasks. ViTs are more flexible than CNNs in their ability to learn information through self-attention mechanisms. ViTs are not only able to learn features from nearby image pixels (in the form of patches), but also learn features from distant image pixels/patches within the image that contribute to the overall image classification label. ViTs will be useful to learn and understand the significance of distal patterns within larger image regions^43,44^.

Furthermore, the incorporation of ensemble methods which function to combine the strengths of multiple different AI models are likely to enhance predictive accuracy and robustness^45,46^. Incorporating a wider range of patient variables may also enhance the models’ predictive power and relevance. Specifically, the inclusion of radiomic features, genomic and proteomic data such as gene expression profiles and cellular biomarkers may provide assistance in recognizing the molecular signatures that are associated with the presence or likelihood of LNM^47^. Evaluating these models in an array of settings and diverse patient populations is critical in ascertaining the real-world applicability of these models. Design and delivery of larger scale, multicentre studies applying these models prospectively on diverse cohorts will assist in the validation process. To facilitate this, the use of federated learning and swarm learning approaches presents opportunities to provide high-quality data for training without the necessity to exchange personal identifiable information about patients, which avoids practical and legal issues surrounding data sharing and data sovereignty for institutions. Federated learning involves several AI models trained on computers independently from each other using separate data sets. During training, model updates are fed-back to a centralized server which updates the model's parameters (weights and biases) based on all the data sets and passes the updated, learned model parameters in a coordinated manner back to participants to continue with their AI model training and validation processes^43,48^. Swarm learning differs from federated learning in that several parties can co-train an AI model together through a coordinated blockchain-based communication. This approach allows exchanging AI model updates directly amongst peers without the need for passing data through a controlled, centralized server. Several advantages are provided with the use of Swarm learning, first, the AI model is not affected through the loss of a single party training the AI model. Second, control of the AI model development does not solely lie with a single party but is distributed amongst all parties. Third, data security and privacy is preserved at a local site. Finally, Swarm learning promotes equality by facilitating data sharing and collaboration amongst research parties^43,49^.

This review has several limitations that should be acknowledged. As all of the studies included in this review were retrospective, there is potential for selection bias due to reliance on pre-existing data of a study population that may not be representative of the broader population. There is also a lack of control over the quality of the data that was obtained and confounding variables, as well as temporal ambiguity when establishing the correct temporal relationship. There was significant heterogeneity in the AI models used to predict LNM and there was a wide range of years the data was collected from; collectively, conclusions drawn from these results need to be interpreted with caution. The restriction to articles only written in the English language could also introduce bias through the omission of possibly relevant articles that were excluded if they were written in another language.

Overall, the application of AI in predicting LNM in early-stage CRC has shown significant promise. Risk evaluation of LNM in CRC has critical implications in guiding therapeutic management and reducing overtreatment of early-stage disease. Further research with larger, multicentre prospective studies inclusive of diverse populations is essential to further ascertain and validate the predictive potential of AI in this setting.

## Supplementary Material

zrae033_Supplementary_Data

## Data Availability

This systematic review synthesized findings from previously published studies. The data supporting the conclusions of this review are available in the original articles cited in the references. Any additional analyses, tables or figures derived from these studies for the purpose of this review are available from the corresponding author upon reasonable request.
